# Reconstructing gene regulatory networks of biological function using differential equations of multilayer perceptrons

**DOI:** 10.1186/s12859-022-05055-5

**Published:** 2022-11-24

**Authors:** Guo Mao, Ruigeng Zeng, Jintao Peng, Ke Zuo, Zhengbin Pang, Jie Liu

**Affiliations:** 1 Science and Technology on Parallel and Distributed Processing Laboratory, National University of Defense Technology, Deya Road, Changsha, 410073 China; 2 Laboratory of Software Engineering for Complex System, National University of Defense Technology, Deya Road, Changsha, 410073 China

**Keywords:** Fully connected neural network, Biological function, Differential equations, Dynamical systems, Link knockout

## Abstract

**Background:**

Building biological networks with a certain function is a challenge in systems biology. For the functionality of small (less than ten nodes) biological networks, most methods are implemented by exhausting all possible network topological spaces. This exhaustive approach is difficult to scale to large-scale biological networks. And regulatory relationships are complex and often nonlinear or non-monotonic, which makes inference using linear models challenging.

**Results:**

In this paper, we propose a multi-layer perceptron-based differential equation method, which operates by training a fully connected neural network (NN) to simulate the transcription rate of genes in traditional differential equations. We verify whether the regulatory network constructed by the NN method can continue to achieve the expected biological function by verifying the degree of overlap between the regulatory network discovered by NN and the regulatory network constructed by the Hill function. And we validate our approach by adapting to noise signals, regulator knockout, and constructing large-scale gene regulatory networks using link-knockout techniques. We apply a real dataset (the mesoderm inducer Xenopus Brachyury expression) to construct the core topology of the gene regulatory network and find that Xbra is only strongly expressed at moderate levels of activin signaling.

**Conclusion:**

We have demonstrated from the results that this method has the ability to identify the underlying network topology and functional mechanisms, and can also be applied to larger and more complex gene network topologies.

## Background

The growth and development of organisms and their responses to internal and external stimuli are controlled by complex internal regulatory mechanisms, including the gene level. Gene regulation network is the mapping of complex regulation mechanism in organism at gene level. At the molecular level and in the microscopic domain, the function of genes is understood as the interaction behavior of complex networks. Cell function is controlled by the interconnections between gene expression mechanisms and gene regulation. The mapping between gene interactions and functions is one of the main research topics in systems biology [1].

Cellular networks undergo steady-state or oscillatory stimulation signals, which provide a way to reconstruct network topology. To understand how the interrelationships of genes in living organisms respond accurately to external signals and perform their functions robustly. For example, the adaptive function of cells [2] refers to the ability of the system to respond to signal changes and return to the pre-stimulated level, which is the key for living systems to perceive large-scale changes [3]. The transient nature of this stimulus response is important to prevent cells from experiencing uncontrolled proliferation or apoptosis [4]. For example, nuclear enrichment of MAP kinase Hog1 completely adaptes to changes in external osmotic pressure and is robust to very low signal fidelity and operating noise [5].

In the construction of small networks, enumeration search [6] has obvious effect on listing all possible network topology modules, but in larger and more complex networks, enumeration method is difficult to calculate. At present, the models used for gene regulation network modeling mainly include the following: Boolean network, Bayesian network, differential equation, etc [7]. Boolean network is a relatively simple model, and the simulation of the system is fixed and relatively rough; Bayesian network is a probabilistic model that can quantitatively and randomly describe the control network; Differential equations can quantitatively and accurately predict the system behavior; Modeling and reconstructing gene regulatory networks from time series data, most of the existing methods [8–12] are based on ordinary differential equations (ODE).

Ordinary differential equation models include linear differential equations and nonlinear differential equations. Linear differential equation models have been used to infer large-scale gene regulatory networks due to their simple structure and few parameters and expression data. For example, Matsumoto et al. [13] proposed the SCODE algorithm based on linear ordinary differential equations to study gene regulatory network information related to the process of cell differentiation. They first performed single-cell sequencing on individual cells, and then used the algorithm to assess differences in expression patterns between individual cells. Aubin et al. [14] proposed the GRISLI method that infers a velocity vector fields in the space of scRNA-seq data from profiles of individual cells, and models the dynamics of cell trajectories with a linear ordinary differential equation to reconstruct the underlying GRN with a sparse regression procedure. Although linear regulatory functions can describe network regulatory system, gene regulatory networks are mostly nonlinear. Many classical nonlinear differential equations that conform to the laws of biochemistry have been proposed to infer GRN, such as S-system model [15], Hill function. In recent years, the S-system model has been widely utilized to infer GRN and biochemical reactions, which follows the theory of a biological system [16], Since the structure of the S-system model is fixed, heuristic search algorithms have been used to search for the optimal parameters of the S-system model. , such as differential evolution (DE) [17], cooperative coevolutionary algorithm [18], sensitivity-based incremental evolution method [19], bat algorithm (BA) [20], immune algorithm (IA) [21], firefly algorithm [22], dissipative particle swarm optimization (DPSO) [23], cockroach genetic algorithm (CGA) [24], hybrid algorithm based on genetic algorithm (GA) and PSO [25].

Hidde De Jong [7] proposed the Hill function as a regulatory function. Hill functions are considered suitable for building GRN models with ODEs [7, 26]. They can quantify activation and inhibition effects of genes. The regulating function can also be sigmoid function [27] commonly used in neural networks, referred to as S-type function, whose input and output characteristics are usually expressed by logarithmic curve or tangent curve. It introduces the necessary nonlinearity and defines an upper bound on the rate of change in molecular concentration. The advantage of this neural network-based differential equation model is that a large number of effective learning algorithms have been developed for the learning of parameters in the regulatory network. For example, Mattias Wahde [28] provided a differential equation system based on feedback neural network, the regulation function is the commonly used logarithmic Sigmoid activation function, and the parameter estimation adopts genetic algorithm. Shen et al. [29] believed that deep learning could be used to search network topology more effectively and train deep neural network to find satisfactory network topology by relying on trajectory and error. The idea is to learn differential equations in data, i.e. use a neural network to train an accurate potential unknown dynamical system [30, 31], the model is difficult to be extended to a large number of genes, so it is difficult to describe the complex behavior of the system.

**Fig. 1:**
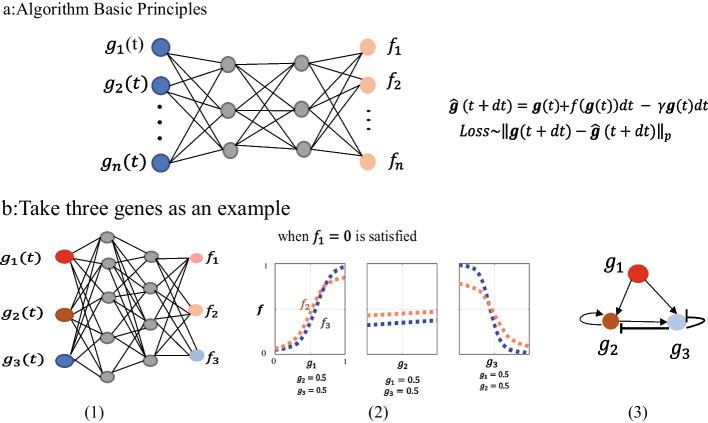
Fully linked neural network. **a** Schematic diagram of the fully connected neural network training synthetic term f. **b** Three genes are used as an example to illustrate. The synthetic term $f_2$($f_3$) of $g_2$($g_3$) is evaluated by a fully connected network. $f_2$ and $f_3$ (wheat and light blue) can depend on all three variables: $g_3$, $g_2$ and the input signal $g_1$

Inspired by the above methods, we propose a multi-layer perceptron-based differential equation method, which specifically transforms the gene regulation network(GRN) system into an input-output regression problem, where the input is gene expression data and the output is the derivative estimated from the expression data. Our method utilizes time-series gene expression data to train a regulatory function that simulates the transcription rate of a gene, which is a fully connected neural network(NN) with a four-layer structure. The fully connected neural network is trained by using the gene expression of the previous moment to predict the gene expression of the next moment, and using the loss function between the obtained prediction result and the real gene expression for feedback training. After training the model, the link knockout technique is used to set the expression value of a gene to 0 and determine the regulatory relationship between genes by looking at the influence of the gene on the synthesis rate (see Materials, Methods and Results for a detailed description). Figure 1a illustrates the detailed work of the overall framework. Figure 1b is used as an example to fully understand the composition of the regulatory relationship between the three genes. The control variable method is used to obtain the relationship between the synthesis rate and the gene over time. When the synthesis rate of gene 1 is restricted to 0, That is, taking gene 1 as the stimulus signal, looking at the changes of the three genes and their corresponding synthesis rates over time, and obtaining the final regulatory relationship between individual genes through the cross-sectional view of the fully connected neural network.

In this paper, we verify whether the regulatory network constructed by the NN method can continue to achieve the expected biological function by verifying the degree of overlap between the regulatory network discovered by NN and the regulatory network constructed by the Hill function (HF). Moreover, our method is verified by three cases: adaptive noise signal, link knockout, and using link knockout technology to build large-scale gene regulatory network. And apply the real dataset (the mesoderm inducer Xenopus Brachyury (XBra) expression) to construct the core topology of gene regulatory network. The resulting network topology can be intuitively explained by the concentration changes between genes, and many target functions can be achieved by comparing the resulting network with existing biological networks.

## Results

### Simulation studies

In this section, we conduct some simulation studies to empirically evaluate our proposed framework under different settings. In what follows, we demonstrate our proposed ability to construct gene regulatory networks in three scenarios: One is a regulatory network that can adapt to the influence of Gaussian white noise, and the other is the simulation of link knockout. The regulatory network obtained by training NN is redundant, and the core gene regulatory network can be obtained by link knockout. The last is to use linked knockouts to construct large-scale gene regulatory networks.

### Case one: adaptation

Since adaptive systems often operate in noisy environments, exploring the adaptive properties and noise immunity of the network is the main goal of this section. It can be seen from Fig. 1 that the fully connected neural network (NN) transfers hidden information from the previous time point to the next time point, and we ask the output node $g_3$ to perform the adaptation function (Fig. 1b). The input node $g_2$ has no functional limitation, but can play an adjustment role. The input node $g_1$ is used as the input signal *I*.

**Fig. 2:**
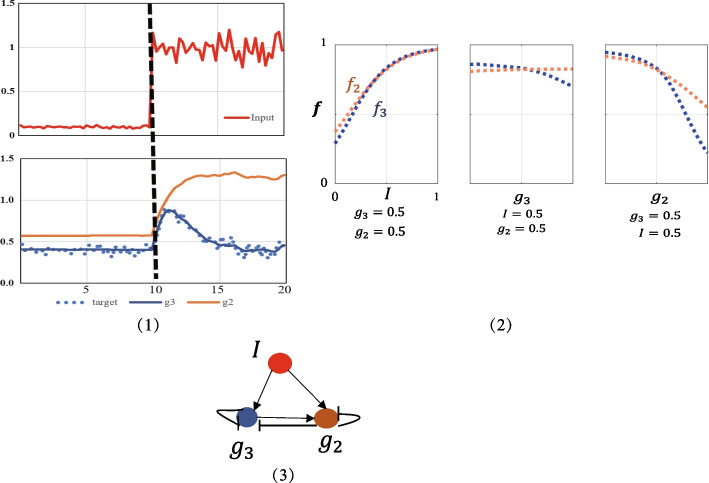
Time evolution process under noisy conditions. (1) Under the stimulus after adding Gaussian white noise to the input signal (the red line of Input), without any constraints on g_2_, the time evolution curves g_3_ and g_2_ obtained after training the NN, and the expression level of g_3_ (blue line) is the same as The target time progress value (target’s blue dotted line) basically matches. (2) Cross-section information obtained by training a NN under noisy conditions. Three panels show the dependence of $f_3$, $f_2$ on *I*, $g_3$ or $g_2$ with the other two variables fixed. (3) The regulatory network obtained from (2)

Therefore, as shown in Fig. 2(1), the time evolution of the input signal (*I*) after adding noise, the expression level of $g_3$ (blue line) is basically consistent with its target time course value (blue line) Dotted line), with a fast response phase and a slower recovery phase. And it can be intuitively seen in Fig. 2 that $f_3$ and $f_2$ change with the increase or decrease of the input $g_3$, $g_2$, *I*, it is easy to know the adjustment logic hidden in NN, and directly construct Gene regulatory network (Fig. 2(3)). Our method achieves a reliable response to Gaussian white noise.

In the context of biological regulatory networks, we need to verify whether the resulting network is biologically feasible. Here, in order to check whether the network obtained by the NN model (Fig. 1b) is reliable, the *f* term in Equation 2 is represented by the hill function, which is widely used in biological regulation modeling. The obtained gene regulatory network (Fig. 1b(3)) can be successfully transferred to the hill function model (Table 1), and the expected adaptive function can be achieved. Similarly, The obtained gene regulatory network (Fig. 2(3)) can be successfully transferred to the hill function model (Table 2).

**Table 1:** Parameters for Hill function model with the topology of Fig. 1b(3) (Hill coefficient $n = 2$)

Link	Activition/Inhibition	b	K
$g_1$ to $g_3$	Act.	4.242	1.198
$g_1$ to $g_2$	Act.	0.691	0.708
$g_2$ to $g_3$	Act.	0.496	0.664
$g_2$ to $g_2$	Act.	1.499	1.300
$g_3$ to $g_2$	Inh.	–	0.166
$g_3$ to $g_3$	Inh.	–	0.231

**Table 2:** Parameters for Hill function model with the topology of Fig. 2(3)(Hill coefficient $n = 2$)

Link	Activition/Inhibition	b	K
*I* to $g_3$	Act.	2.148	1.573
*I* to $g_2$	Act.	0.161	0.091
$g_3$ to $g_2$	Act.	0.178	0.051
$g_3$ to $g_3$	Inh.	–	1.977
$g_2$ to $g_3$	Inh.	–	0.976
$g_2$ to $g_2$	Inh.	–	1.278

### Case two: link-knockout knockout

**Fig. 3:**
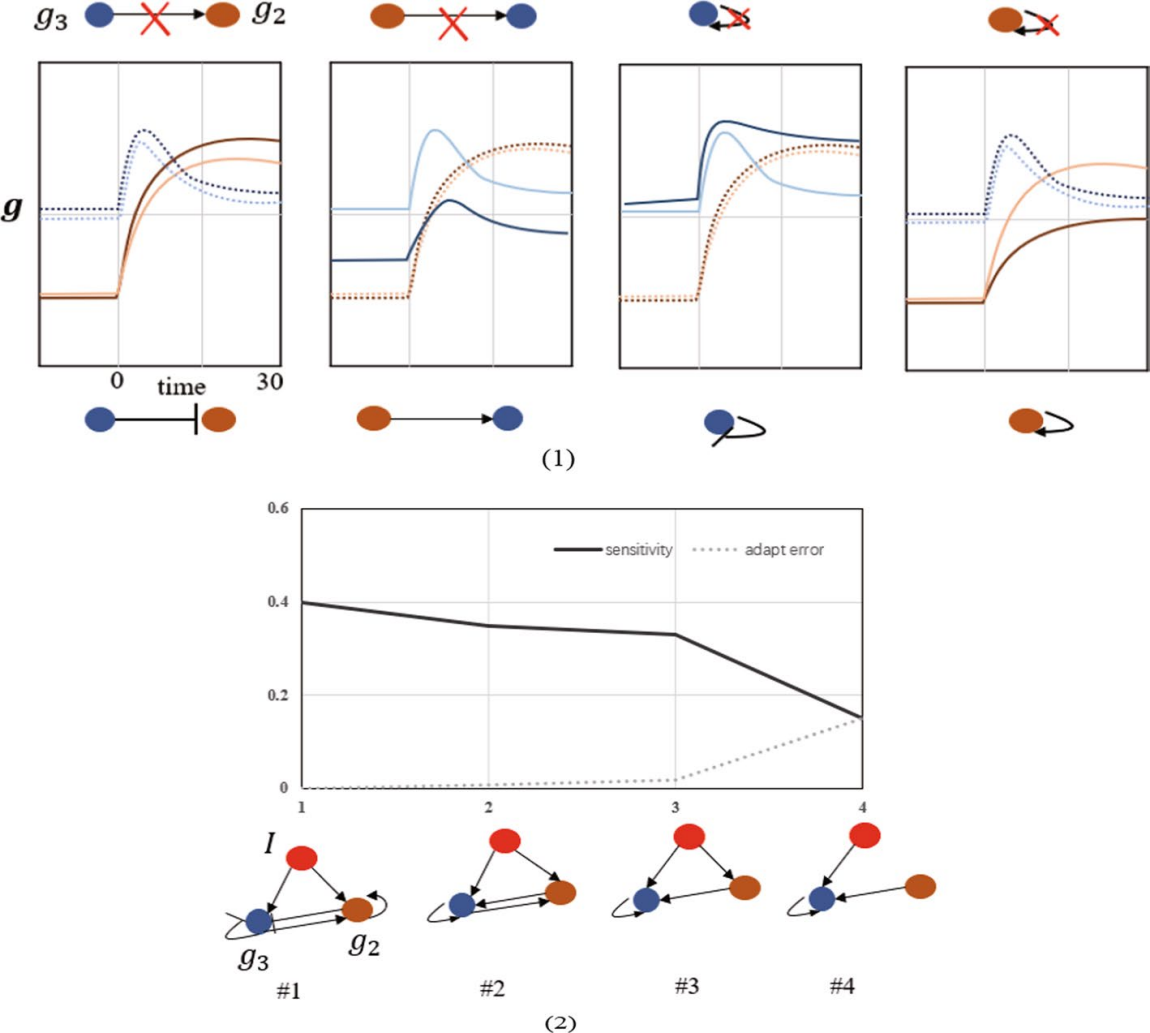
Simulate regulator knockout. (1) The perturbed *f* function can be iterated to simulate the effect of mutants in which specific regulatory chains are deleted. For example, deletion of $g_3$’s modulating effect on $g_3$ leads to an increase in $g_3$ (from darker to brighter solid green lines), indicating self-inhibition (shown in the third panel). The difference in $g_2$ levels is not important here (dotted line). A similar argument applies to the other three panel. (2) Describes the sensitivity of network sequence and adapt to the error. Pane shows the knockout technology through links, step 1 to 4 of the evolution process of the network topology. The minimum incoherent feed forward motif appears naturally (topology #4), before the network has too few links to adapt

According to Formula 5, the regulation logic of the gene regulatory network can be obtained by knocking out the difference before and after gene expression obtained by a certain edge, such as knocking out the regulatory link from $g_3$ to $g_2$, by setting $g_3$ to 0 when calculating $f_2$. Four examples of link knock-out with $\lambda$ set to 0 are shown in Fig. 3(1). The first panel shows that $g_3 = 0$ is entered into NN to obtain the value of $f_2$. $g_2$ expression level increases from the lighter orange line to the brighter orange line, indicating that $g_3$ inhibits $g_2$. The second panel shows that $g_2$ is set to 0 and input into NN to obtain $f_3$. In the figure, the expression level of $g_3$ decreases from lighter blue to darker blue, indicating that $g_2$ stimulates $g_3$. In the third panel, $g_3$ was set to 0 and input into NN to obtain $f_3$. The expression level of $g_3$ increased from lighter blue to darker blue, indicating $g_3$ self-inhibition, and in the same way, the fourth panel showed $g_2$ self-activation.

As above mentioned in this paper, through the order to remove unnecessary link to adjust repeatedly sparse network. The Fig. 3(2) depicts the sensitivity(response peak) and adaptive error(difference between the pre-stimulus and the fully adapted $g_3$ levels) of the network sequence. The network shown in Panel #1 in Fig. 3(2) includes a basic adaptive function: incoherent feed-forward loops. #1 is the regulatory network learned by NN without any constraints, which has redundancy. By linking knockout technology, applications to the existing links knockout, find the smallest change after deleting network, links to knock out after retraining within NN can get effective gene regulation network of sparse #4 (in sensitivity and adaptation error is equal). #4 is the adaptive function with the least links to achieve the minimum incoherent feedforward network.

### Case three: Large-scale gene regulation networks are constructed using link knockout techniques

To apply to large-scale data, we evaluate the performance of our model using two datasets, each containing time-series expression profiles. Time-series data reflects how the network responds to perturbations and how it recovers after the perturbations are removed. The first one is the simulation data, we choose the InSilico_Size100 dataset from the DREAM4 In Silico Network Challenge [32]. The second one is from the real dataset, we select a large-scale E. coli dataset (GSE20305) from the Gene Expression Omnibus (GEO) database [33]. The gold standard benchmark for E.coli consists of part from DREAM5 challenge [34] and other experimentally verified part from RegulonDB [35]. GSE20305 [33] provides real gene time-series data of E. coli under different experimental environments. We choose the data under the three conditions (cold stress, heat stress and oxidative stress) to make up our experimental dataset. The specific information of each dataset is shown in Table 3.

**Table 3:** The description of datasets used in experiments

DataSet	Network	Number of genes	Number of TFs	Number of samples	Time points	edges	Density
DREAM4 InSilico_Size100	Network_1	100	100	10	21	176	0.0176
DREAM4 InSilico_Size100	Network_2	100	100	10	21	249	0.0249
DREAM4 InSilico_Size100	Network_3	100	100	10	21	195	0.0195
DREAM4 InSilico_Size100	Network_4	100	100	10	21	211	0.0211
DREAM4 InSilico_Size100	Network_5	100	100	10	21	193	0.0193
E.coli	Network_1	1484	163	3	8	3080	0.0127

To evaluate the effectiveness of our method on the datasets in Table 3, Serveral methods are chosen as baselines as follows:

- GENIE3 [36]: an approach to infer gene regulatory networks from gene expression data. It trains a random forest model that predicts the expression of each gene in the dataset and uses the expression of transcription factors (TFs) as input.
- BiXGBoost [37]: it is a bidirectional-based method by considering both candidate regulatory genes and target genes for a specific gene. Moreover, BiXGBoost utilizes time information efficiently and integrates XGBoost to evaluate the feature importance.
- SIGNET [38]: a deep learning-based framework for capturing complex regulatory relationships between genes under the assumption that the expression levels of transcription factors participating in gene regulation are strong predictors of the expression of their target genes.
- GNIPLP [39]: an approach to infer GRNs from time-series or non-time-series gene expression data. GNIPLR projected gene data twice using the LASSO projection (LSP) algorithm and the linear projection (LP) approximation to produce a linear and monotonous pseudo-time series, and then determined the direction of regulation in combination with lagged regression analyses.
- PoLoBag [40]: it is an ensemble regression algorithm in a bagging framework where Lasso weights estimated on bootstrap samples are averaged. These bootstrap samples incorporate polynomial features to capture higher-order interactions.

For a fair comparison of the above methods in this experiment, we always use the default parameters when running the program. We systematically evaluate the model using seven evaluation metrics, namely True Positive Rate(TPR), False positive rate(FPR), Matthews correlation coefficient (MCC), Accuracy(ACC), F-measure(F1), Area Under the Receiver Operating Characteristic curve(AUROC), Area under the precision-recall curve (AUPR). Experimental results for each method provide all predicted edges and their corresponding weights. The higher the weight, the higher the credibility of the regulatory relationship. Since different thresholds construct different GRN, the FPR, TPR, MCC, ACC and F1 measures are also correspondingly different.

**Fig. 4:**
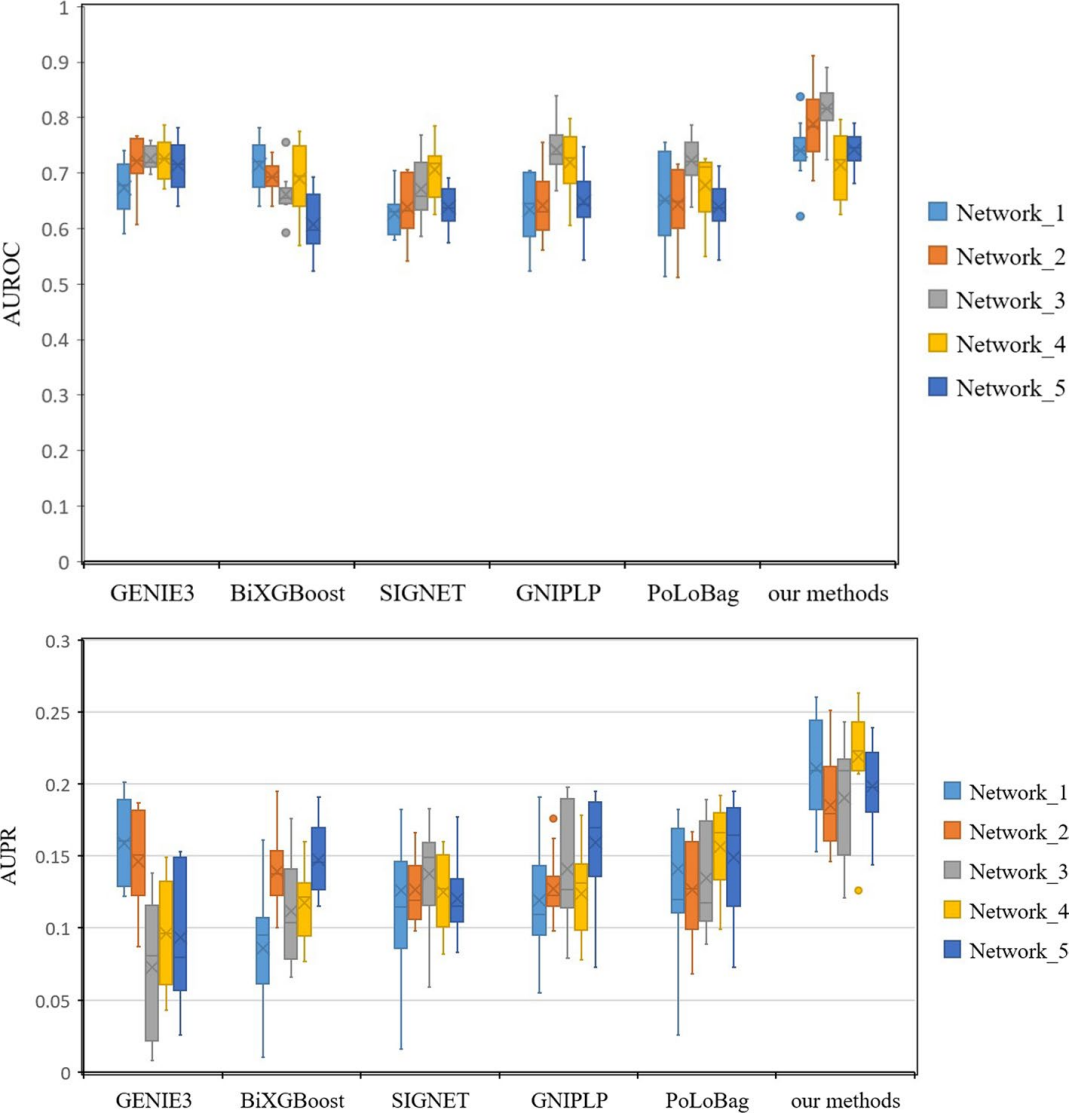
The AUROC and AUPR of GENIE3, BiXGBoost, SIGNET, GNIPLP, PoLoBag and our methods on DREAM4 InSilico_Size100 five networks

The experiments are first performed on the DREAM4 InSilico_Size100 five networks. The edge weights predicted by all methods are sorted, and the first 250 predicted values are set to 1, and the other predicted values are set to 0. The following five indicators are calculated as shown in Table 4. The results in Table 4 show that our method outperforms the comparative methods, which indicates that our method can construct regulatory networks of time-series gene expression data by linking knockout techniques. In order to comprehensively consider the experimental results under different thresholds, we choose AUROC and AUPR values as evaluation criteria. Due to the randomness of the NN, the results will be different from run to run. In our experiments, these methods are ran 10 times and the results are presented in Fig. 4. For Network 4, the GENIE3 method outperforms the rest on AUROC. In InSilico_Size100 Networks 1–3 and 5, our method has higher average AUROC, and the average AUPR number on Networks 1–5 is better than other methods.

**Table 4:** Evaluating GRN inferring methods on DREAM4 InSilico_Size100

Method	TPR	FPR	MCC	ACC	F1
Network_1					
GENIE3	0.098	0.001	0.232	0.981	0.168
BiXGBoost	0.226	0.006	0.310	0.976	0.302
SIGNET	0.190	0.005	0.288	0.976	0.268
GNIPLP	0.180	0.004	0.286	0.966	0.218
PoLoBag	0.215	0.006	0.307	0.979	0.298
Our methods	0.392	0.007	0.342	0.98	0.348
Network_2					
GENIE3	0.134	0.004	0.241	0.972	0.201
BiXGBoost	0.182	0.006	0.263	0.964	0.239
SIGNET	0.210	0.006	0.274	0.937	0.293
GNIPLP	0.234	0.007	0.284	0.945	0.294
PoLoBag	0.253	0.007	0.294	0.937	0.295
Our methods	0.321	0.007	0.312	0.956	0.303
Network_3					
GENIE3	0.104	0.003	0.239	0.962	0.227
BiXGBoost	0.178	0.005	0.243	0.943	0.213
SIGNET	0.21	0.007	0.279	0.952	0.288
GNIPLP	0.183	0.006	0.273	0.913	0.302
PoLoBag	0.174	0.005	0.251	0.929	0.291
Our methods	0.227	0.007	0.322	0.943	0.316
Network_4					
GENIE3	0.172	0.004	0.238	0.937	0.206
BiXGBoost	0.211	0.007	0.281	0.923	0.291
SIGNET	0.193	0.006	0.293	0.953	0.273
GNIPLP	0.199	0.005	0.301	0.947	0.285
PoLoBag	0.214	0.007	0.293	0.932	0.293
Our methods	0.247	0.008	0.362	0.974	0.382
Network_5					
GENIE3	0.143	0.005	0.263	0.983	0.194
BiXGBoost	0.175	0.003	0.271	0.955	0.237
SIGNET	0.193	0.006	0.289	0.932	0.283
GNIPLP	0.163	0.003	0.284	0.925	0.249
PoLoBag	0.175	0.004	0.291	0.943	0.302
Our methods	0.203	0.007	0.320	0.949	0.319

**Fig. 5:**
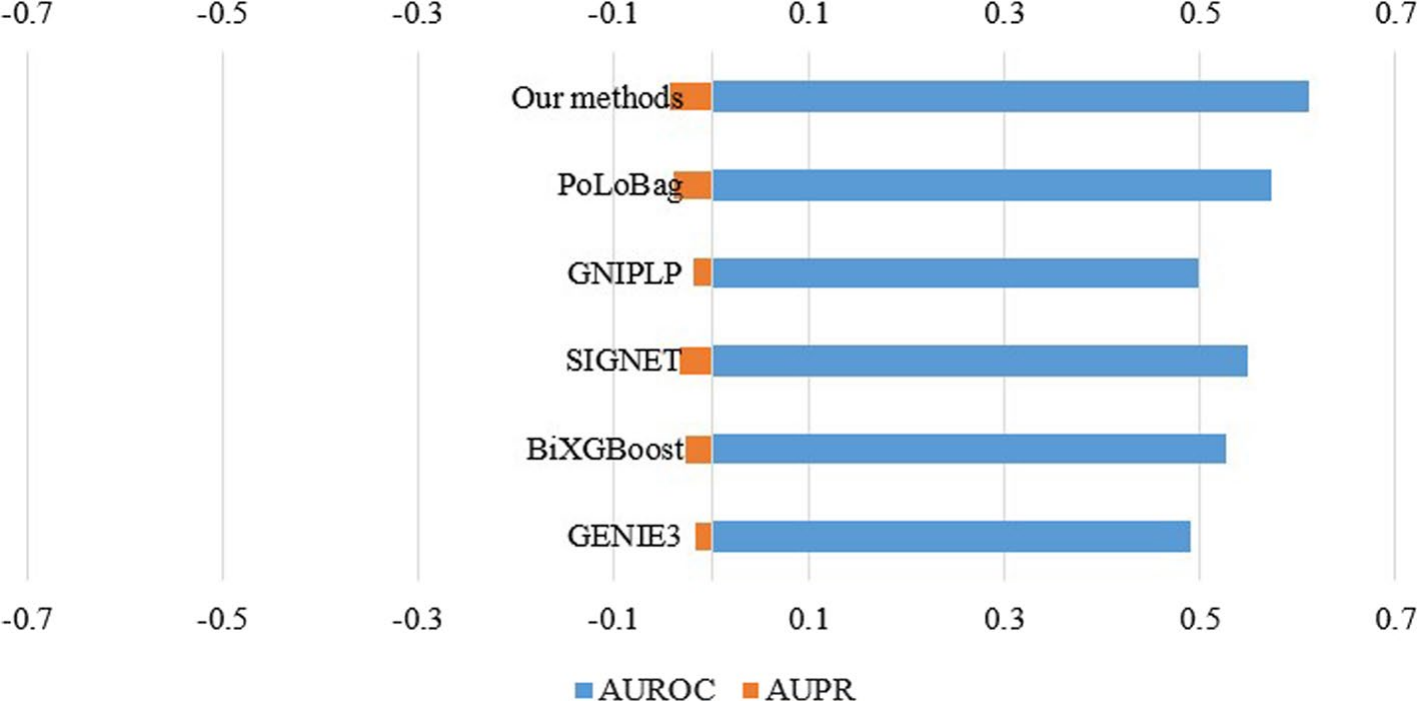
*E. coli* network including 1484 genes. Each bar represents the performance of one method in which the abscissas are the corresponding AUROC (right) and AUPR (left) values

For the E.coli dataset, we sorted the edges predicted by all methods and set the predicted value of the first 3080 edges to 1, and the value of the other predicted edges to 0. The FPR, TPR, MCC, ACC, and F1 measures are calculated between the predicted labels and the ground truth labels. The results are shown in Table 5. As shown in Table 5, Our methods perform the best. In order to consider the case of different thresholds, we show the results of ten average runs of all methods in Fig. 5. In particular, all available methods obtain worse results with less than 0.05 AUPR values on E.coli network (Fig. 5). This is due to the fact that AUPR tends to present smaller values on large-scale networks. Compared with the other five methods, our method achieves the best AUROC and the best AUPR. To test the efficiency of our method, we compare the running time of the six methods on a 32GB RAM, Intel(R) Xeon(R) CPU E5-2630 computer. The comparison results on the DREAM4 InSilico_Size100 and E.coli datasets are shown in Table 6. The table shows the average running time values of the six algorithms executed 10 times. Our method is relatively faster than other state-of-the-art methods.

**Table 5:** Evaluating GRN inferring methods on E.coli

Method	TPR	FPR	MCC	ACC	F1
GENIE3	0.142	0.024	0.086	0.970	0.083
BiXGBoost	0.183	0.028	0.118	0.951	0.134
SIGNET	0.191	0.027	0.132	0.956	0.151
GNIPLP	0.162	0.024	0.103	0.97	0.102
PoLoBag	0.204	0.026	0.142	0.934	0.169
Our methods	0.367	0.025	0.275	0.961	0.286

**Table 6:** The running time comparison of the algorithms

DataSet	GENIE3	BiXGBoost	SIGNET	GNIPLP	PoLoBag	Our methods
DREAM4 InSilico_Size100	10 min 36 s	9 min 10 s	11 min 3 s	9 min 43 s	10 min 23 s	8 min 52 s
*E. coli*	5 h 55 min	4 h 45 min	6 h 13 min	5 h 45 min	6 h 11 min	5 h 7 min

### Application to the Xenopus Brachyury (XBra)

In this section, real data are used to demonstrate the effectiveness of our method in a complex situation—the Activin/GSC/Xbra System. This is a well-researched system, including experiments and modeling [41]. Here, the fully connected data network model is used to solve the inverse problem, finding the core gene regulatory network given the observed gene expression of the Xenopus Brachyury as the desired output. The results obtained by NN were compared with known biological networks.

**Fig. 6:**
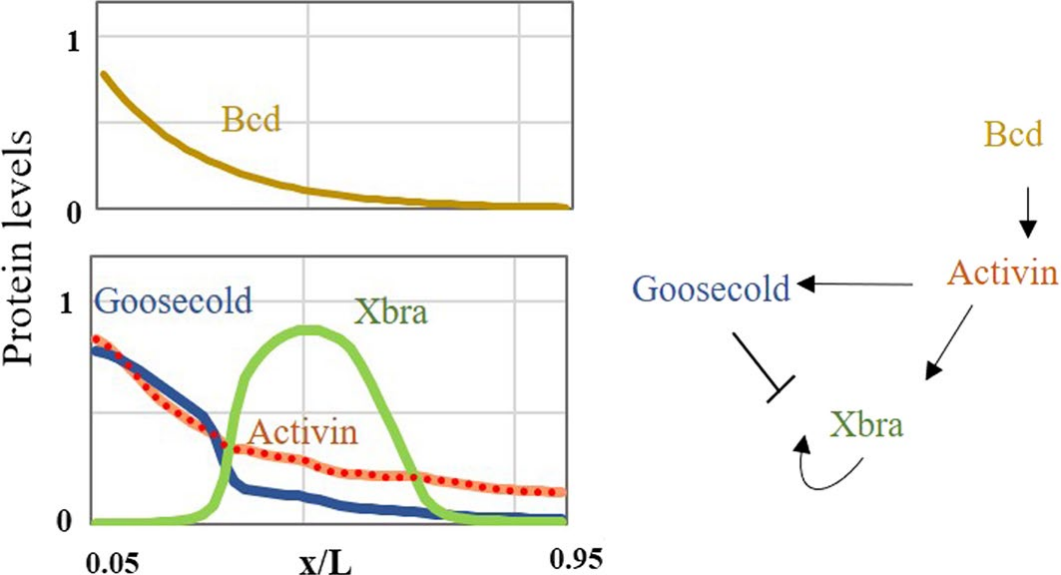
the activin/gsc/Xbra system. The Activin gene was activated by the input signal of morphogenetic gradient (Bcd), so it began to imitate its gradient mode. The Activin gene activated Xbra gene and opened the positive feedback of Xbra gene at a certain threshold. The Activin gene activates the Goosecold gene, and when the concentration of the two genes accumulates high enough, it forces the Xbra gene down. However, the concentration is highest only on the left side, when the concentration of Goosecold gene is low and its inhibitory effect is low, so that Xbra gene reaches a stable state

Gene regulation in Fig. 6 follows the NN modeling in Fig. 1a, where *g* and *f* are three-dimensional vectors (Activin, Goosecold, Xbra) and input signal $g_1$(Bcd). The expression of three genes is taken from the study of Green et al. [41], and the morphogenetic gradient (Bcd) is regarded as static. The results of 40 repetitions of NN training were all overlapped with the target graph (as shown in Fig. 6). When using the link knockout method, the gene regulatory network obtained is consistent with the known network structure. In Tables 7, the frequency with which the link is activated, non-existent, and inhibited by 40 repetitions of training is listed, and in accordance with the majority coloring (orange activated, blue inhibited), the majority network (Fig. 6 on the right) has a very similar structure to the known biological network revealed in experiment [41]. This experiment helps demonstrate the effectiveness of our method on real data.

**Table 7:** Statistical data of gene regulation network obtained by 40 repetitions of training NN

From	To		
	Goosecold	Activin	Xbra
Bcd	0/38/2	**40/0/0**	4/36/0
Goosecold	0/30/10	1/38/1	*0/1/39*
Activin	**39/0/1**	0/40/0	**32/8/10**
Xbra	5/35/0	0/40/0	**36/0/4**

Known biological interactions **Act.**/Null/*Inh.*

## Discussion

In this paper, we propose a multi-layer perceptron-based differential equation method, which operates by training a fully connected neural network (NN) to simulate the transcription rate of genes in traditional differential equations. From the dataset validation results, our algorithm is superior to other methods, and its good performance is attributed to the use of neural networks to simulate unknown dynamical systems. This has many advantages. First, there is no detailed mathematical equation format for using the input-output function of a multilayer perceptron. Training a neural network is to establish the necessary logical connections between input and output nodes, without specific constraints. Second, fully connected neural networks can speed up model training and scale to large-scale complex gene regulatory networks. Finally, neural networks are well suited for building gene regulatory networks on time-series gene expression data due to their limited short-term memory advantage.

Our goal is to visually explain how gene regulatory networks (GRNs) achieve concentration-dependent responses. However, the number of different mechanisms that may exist in cells, such as feedback or local cell-cell communication, is unclear. Some well-defined biological functions may have broad kinetic interpretations (even for relatively simple three-gene networks and limited forms of modeling). There are more complex cellular processes that cannot simply be attributed to activating or repressive regulation. The structure of the regulatory network itself should be re-explored in a more comprehensive context. The method developed here can provide ideas for further exploration of reconstructed gene regulatory networks in the future, and interesting future research topics can apply our method to different real-world biological and biomedical data problems.

## Conclusions

A long-standing question in biology is how complex biological networks perform complex regulatory functions. One strategy is to exhaustively search all possible biological networks for single or multiple functions, which is only suitable for small gene networks. For a biological network of four genes, the computational complexity of the exhaustive search method is enormous. In this study, we propose a multi-layer perceptron-based differential equation method. Figure 1a illustrates the specific work of the whole framework. Our method utilizes time-series gene expression data to train a regulatory function that simulates the transcription rate of a gene, which is a fully connected neural network (NN) with a four-layer structure. The fully connected neural network is trained by using the gene expression of the previous moment to predict the gene expression of the next moment, and using the loss function between the obtained prediction result and the real gene expression for feedback training. After the model is obtained after training, the link knockout technique is used to set the expression value of a gene to 0, and the regulatory relationship between genes can be judged by looking at the effect of the gene on the synthesis rate.

First we verify the adaptation function of our method. The adaptive function is performed by training a NN, and our method also performs well in the presence of Gaussian white noise on the internal and external stimulus signals. Then, through the link knockout technique, redundant links are eliminated from the gene regulatory network trained by NN, and an effective core gene regulatory network is finally obtained. Finally, to validate our approach on large-scale datasets, we use InSilico_Size100 time series simulation data and E.coli real datasets. Our model is compared with three state-of-the-art regression models on these two datasets. Experiments show that our method performs well in all six networks, which proves the good scalability and adaptability of our method. In addition to validating on a large-scale real dataset, we also validate our method on a real dataset (Xenopus laevis) with five genes to demonstrate its effectiveness. Our method can help discover the regulatory logic and network topology of complex tasks. For the resulting network topologies, it is possible to intuitively explain how their structures generate their functions, thus linking network topology to function.

## Methods

### Nonlinear ordinary differential equation models

In the gene regulation system, the time effect variable xi is used to represent the expression level of the ith gene at time t, and the value of this variable is non-negative. Then, the regulatory relationship between n genes in the system can be expressed by ordinary differential equations: One is a regulatory network that can adapt to the influence of gaussian white noise, and the other is the simulation of link knockout. The regulatory network obtained by training NN has redundancy, and the most core gene regulatory network can be obtained by link knockout. The last is the use of linked knockouts to build large-scale gene regulatory networks.

1 $$\begin{aligned} \begin{array}{c} \frac{dx_i}{dt} = {f_i}({x_1},{x_2},\ldots ,{x_n}),1 \le i \le n. \end{array} \end{aligned}$$

The above equations are also called kinetic equations. where $\frac{dx_i}{dt}$ indicates the rate of change of the expression level of the *i*-th gene at time *t*, ${x_1},{x_2},\ldots ,{x_n}$ represents the expression level of each gene. Therefore, the expression change rate of the *i*-th gene at time *t* depends on the expression levels of other genes, including its own expression level $x_i$. The structure of the function ${f_i}({x_1},{x_2},\ldots ,{x_n})$ on the right-hand side of Equation 1 indicates the internal regulatory mechanism between genes, that is, the structure of the regulatory network.

In most cases, the interactions between genes exhibit complex nonlinear relationships. At this time, the nonlinear regulation function $f_i(x)$ can better explain the real situation in the organism, it is usually considered that the function *f* is a continuously differentiable and monotonically increasing bounded function. Here, we use the hill function to model the complex GRN structure. The dynamics of this GRN can be modeled as:

2 $$\begin{aligned} \begin{array}{c} {f_i} = \left( {\sum \nolimits _j {h_{ij}^ + } } \right) \left( {\prod \nolimits _l {h_{il}^ - } } \right) \end{array} \end{aligned}$$

Here $h_{ij}^ + = \frac{{{b_{ij}}g_j^n}}{{K_{ij}^n + g_j^n}}$ represents the activation item, and $h_{il}^ - = \frac{{K_{il}^n}}{{K_{il}^n + g_l^n}}$ represents the inhibitory item. For simplicity, we set the Hill coefficient $n = 2$ in the enumeration study. Each activation link $h_{ij}^ +$ has two parameters *K* and *b*, while the inhibitory link $h_{il}^ -$ has only one parameter *K*. For each network topology, the network topology is considered ’successful’ when the parameters (*K* and *b*) are sampled independently of the exponential distribution $p(x) = {e^{ - x}}$(refer to the study of Ehsan et al. [42]), 100,000 groups of random parameters are sampled and no less than 2 groups of parameters are obtained. The exhaustive search of hill function model used in this paper is only a verification step, and some false positives do not affect our conclusions.

### Fully connected neural network model

In this paper, multilayer perceptron is used to obtain the gene synthesis rate *f* in time series gene expression data. In biological cells, the regulatory relationship between genes may be time-lag. Therefore, the input layer of multi-layer perceptron in our algorithm is the expression level of all genes at the *t* time point, and the output layer is the synthesis rate *f* of corresponding genes at the *t* time point. In this paper, the activation function of the hidden layer shown in Fig. 1a is ReLU, and the activation function of the output layer is sigmoid, so the value of the synthesis rate *f* is between 0 and 1. They are respectively expressed as:

3 $$\begin{aligned} {\text{ReLU}}(x) = \max (0,{w^T}x + b). \end{aligned}$$

4 $$\begin{aligned} \begin{array}{c} Sigmoid(x) = \frac{1}{{1 + {e^{ - x}}}}. \end{array} \end{aligned}$$

As shown in Fig. 1b using three genes as an example, the ordinary differential equation of the corresponding gene regulatory network is expressed as:

5 $$\begin{aligned} \begin{array}{c} \frac{{d{g_i}}}{{dt}} = {f_i}(g_1,g_2,g_3) - \lambda {g_i};i = 1,2. \end{array} \end{aligned}$$

where the $f_i(g_1,g_2,g_3)$ function contains the regulatory relationship between genes. The Euclidean distance between ${\hat{g}}(t + dt)$ calculated by the ordinary differential equation formula in Fig. 1a and the expression level of gene $g(t + dt)$(time step is 1) at the next moment used as the training loss function of NN, $Loss = \root 2 \of { {\textstyle \sum _{t}}({\hat{g}}(t + dt) - g(t+dt))^{2} }$. $\lambda {g_i}$ represents the degradation term, we simply set $\lambda =1$. In reality the degradation term can be represented by the diagonal term of the synthetic term *f*.

In Fig. 1b, three genes are used as an example to demonstrate that our method can intuitively construct gene regulatory networks. Taking $g_1$ as the stimulus signal, the neural network training principle is shown in Fig. 1b(1), and Fig. 1b(2) represents the cross-sectional information obtained by training the NN. When $f_1=0$ is satisfied, what is shown in Fig. 1b(2) is that $f_2$ (blue dotted line) and $f_3$ (orange dotted line) increase with the increase of $g_1$, that is, $g_1$ activates $g_2$, and $g_1$ activates $g_3$, inhibited by $g_3$ after $g_3$ reaches a steady state. The regulatory network extracted from the information of Fig. 1b(2) is composed of an incoherent feedforward loop, and b(3) is the regulatory network obtained from the cross-sectional information of the neural network of Fig. 1b(2).

### Link knockout technique

For regulatory networks with many more genes, direct visualization of the *f*-function is difficult. Once we have a predictive model between all the genes and the synthetic term *f*, we question which genes in the gene pool have a strong influence on the synthetic term *f*. Therefore, we introduced the linked knockout technique, which passes raw data to the data for gene knockout, i.e., sets the expression of one gene at a time to 0, and uses the expression of the remaining genes as input to predict a specific synthetic term expression status. Therefore, this method can effectively improve the ability of constructing the regulatory network without reading the weight of NN. A disadvantage of this method is that when the synthesis rate of $g_j$ is strongly inhibited by the highly expressed $g_i$ gene. that is, when the expression of gene $g_i$ is set to 0, the value of synthesis rate $f_j$ obtained through neural network training will be very large. Therefore, a more accurate measure of the change in $f_j$ with a fold change in $g_i$ is:

6 $$\begin{aligned} {\Delta _{ij}} = {f_j}({g_1},\ldots ,{g_i}) - {f_j}({g_1},\ldots ,\mu {g_i});0< \mu < 1. \end{aligned}$$

This formula represents the link knockout experiment. $\mu$ represents discount factor, $\mu = 0$ represents link knockout. We truncated the domain where transcription factor *i* binds to gene *j*. With the regulatory link from node *i* to *j* being knocked down by a factor $\mu$, the NN output (synthesis term $f_j$ ) changes accordingly. $\Delta _{ij}$ reflect the regulation effect of $g_i$ on $g_j$. A more intuitive example in Fig. 3 depicting the mutational trajectory where the regulatory link from $g_3$ to $g_2$ is knocked out is given by:

7 $$\begin{aligned} \left\{ {\begin{array}{*{20}{c}} {\frac{{{dg_2}}}{{dt}} = {f_2}(\mu {g_3},{g_2}) - \lambda {g_2}}\\ {\frac{{d{g_3}}}{{dt}} = {f_3}({g_3},{g_2}) - \lambda {g_3}} \end{array} } \right. ;0< \mu < 1 \end{aligned}$$

Figure 3(1) first panel shows that the increase of $g_3$ level means that $g_2$ negatively regulates or inhibits $g_3$, whereas the decrease of $g_3$ level means that $g_2$ positively regulates or promotes $g_3$.

## Data Availability

Our source codes and data can be found in URL:https://github.com/lhfkd/NNGRN. InSilico_Size100 time series data is automatically generated by GeneNetWeaver 2.0 (http://gnw.sourceforge.net/).
